# A Spanish-language translation for the U.S. of the type 2 diabetes stigma assessment scale (DSAS-2 Spa-US)

**DOI:** 10.3389/fcdhc.2022.1057559

**Published:** 2022-12-23

**Authors:** Kevin L. Joiner, Mackenzie P. Adams, Amani Bayrakdar, Jane Speight

**Affiliations:** ^1^ Department of Health Behavior and Biological Sciences, School of Nursing, University of Michigan, Ann Arbor, MI, United States; ^2^ School of Nursing, American University of Beirut, Beirut, Lebanon; ^3^ School of Psychology, Deakin University, Geelong, VIC, Australia; ^4^ The Australian Centre for Behavioural Research in Diabetes, Diabetes Victoria, Melbourne, VIC, Australia

**Keywords:** social stigma, psychometrics, type 2 diabetes, translation, survey

## Abstract

**Background:**

Diabetes stigma is recognized to negatively impact health-related outcomes for people living with type 2 diabetes (T2D), but there is a dearth of evidence among U.S. Latino adults with T2D. Our aim was to develop a Spanish-language translation of the Type 2 Diabetes Stigma Assessment Scale (DSAS-2) and examine its psychometric properties among U.S. Latino adults with T2D.

**Methods:**

The translation was developed through a multi-step process, including a focus group with community health workers (n=5) and cognitive debriefing interviews with Latino adults with T2D (n=8). It was field-tested in an online survey of U.S. Latino adults with T2D, recruited *via* Facebook (October 2018 to June 2019). Exploratory factor analysis examined structural validity. Convergent and divergent validity were assessed by testing hypothesized correlations with measures of general chronic illness stigma, diabetes distress, depressive and anxiety symptoms, loneliness, and self-esteem.

**Results:**

Among 817 U.S. Latino adults with T2D who participated in the online survey, 517 completed the Spanish-language DSAS-2 (DSAS Spa-US) and were eligible for the study (mean age 54 ± 10 years, and 72% female). Exploratory factor analysis supported a single-factor solution (eigenvalue=8.20), accounting for 82% of shared variance among the 19 items, all loading ≥ 0.5. Internal consistency reliability was high (α=0.93). As expected, strong, positive correlations were observed between diabetes stigma and general chronic illness stigma (r_s_=0.65) and diabetes distress (r_s_=0.57); medium, positive correlations, between diabetes stigma and depressive (r_s_=0.45) and anxiety (r_s_=0.43) symptoms, and loneliness (r_s_=0.41); and a moderate negative correlation between diabetes stigma and self-esteem (r_s_=-0.50). There was no relationship between diabetes stigma and diabetes duration (r_s_=0.07, ns).

**Conclusion:**

The DSAS-2 Spa-US is a version of the DSAS-2, translated into Spanish, that has good psychometric properties for assessing diabetes stigma in U.S. Latino adults with T2D.

## Introduction

An estimated 37 million U.S. adults are living with diabetes: the nationwide prevalence is nearly 15%, and the estimated yearly cost is $327 billion (1). Many adults with type 2 diabetes (T2D), accounting for 90 to 95 percent of diabetes, encounter diabetes-related stigma and discrimination in social spaces, workplaces, and healthcare facilities (2–5). Stigma processes occur due to a characteristic that “marks” an individual as different from others, typically in contexts of culture and/or power (6). There are two general forms of stigma: social stigma and self-stigma (6). Social stigma can be experienced and perceived by an individual as blame, judgment, stereotyping, rejection, exclusion, and discrimination. Self-stigma occurs when society’s negative beliefs are internalized by an individual, manifesting as feelings of embarrassment, shame, reduced self-efficacy, and/or reduced self-esteem.

The Type 2 Diabetes Stigma Assessment Scale (DSAS-2) was developed to assess experienced and perceived diabetes-related stigma among adults with T2D (7). Development of the DSAS-2 was informed by a comprehensive literature review and qualitative research (2, 8). Subsequent research using the DSAS-2 in Australian adults with T2D showed that experiences and perceptions of diabetes stigma are associated with higher levels of diabetes distress and more depressive and anxiety symptoms (3). A study using the DSAS-2 in a large sample of U.S. adults with T2D found that diabetes stigma is associated with higher diabetes distress, lower engagement in diabetes self-management, lower diabetes self-efficacy, and lower quality interactions with health care professionals (9).

While medical advancements and new technologies have transformed T2D health and health care over the last few decades, not all people with T2D have benefited equally (10). People from minority racial and ethnic backgrounds are disproportionately affected by T2D, with higher rates of diabetes-related complications and mortality (11). Adults who identify as Hispanic or Latino (hereto forth referred to as Latino) make up the largest ethnic minority group in the U.S., comprising 17.5% of adults (6.5 million people) with diabetes (1). Seventy percent of Latinos in the U.S. speak Spanish at home, and those with limited English proficiency are often excluded from research. This can be due to factors such as researchers not devoting sufficient resources to building trusted relationships with Spanish-speaking Latino populations, lack of professional interpreters, and Spanish-language study materials not being made available (12). Furthermore, research suggests that individuals’ experiences and perceptions of stigma related to chronic illnesses vary by race and ethnicity (13, 14). These deficiencies pose a significant threat to representing Latino adults in diabetes research, addressing disparities, and advancing the healthcare system’s capacity to meet the needs of diabetes disparities populations.

Therefore, the aims of this study were to conduct a cultural and linguistic validation of the DSAS-2, to create a Spanish-language version (DSAS-2 Spa-US), and to assess its psychometric properties in a sample of U.S. Latino adults with T2D. This will enable assessment of diabetes stigma in this population to track and evaluate gaps in health equity.

## Materials and methods

The study included two phases: 1) developing the DSAS-2 Spa-US, and 2) field-testing the DSAS-2 Spa-US in an online survey of Spanish-speaking U.S. Latino adults with T2D. The study was approved by the Health Sciences and Behavioral Sciences Institutional Review Board of the University of Michigan (HUM00139792 and HUM00142346).

### Characteristics of the DSAS-2

The DSAS-2 is comprised of 19 items, which form a total scale (19 items), and three subscales: (a) Treated Differently (six items), (b) Blame and Judgement (seven items), and (c) Self-Stigma (six items) (7). Each item is presented as a statement with five Likert-type ratings ranging: 1 (strongly disagree), 2 (disagree), 3 (unsure), 4 (agree), and 5 (strongly agree). The scale and subscales are scored by summing the relevant items. Higher scores on the total scale are interpreted as more experienced or perceived diabetes stigma, and higher scores on the subscales indicate greater endorsement of experiencing or perceiving being treated differently, experiencing or perceiving blame or judgment, and experiencing or perceiving self-stigma.

### Translation of the DSAS-2 into Spanish for the U.S.

The translation team included the primary investigator, who holds a BA in Spanish and a Ph.D. in nursing, and three translators, who were doctoral-prepared or held a Ph.D. in Spanish. Two translators identify as Latino and are native Spanish speakers, one of Mexican heritage, and one of Peruvian heritage. One translator identifies as non-Hispanic White and is a native English speaker from the U.S. In the first step, the translators who are native Spanish speakers independently translated the DSAS-2 from English into Spanish, which resulted in two initial prototypes of the DSAS-2 Spa-US. In the second step, the team compared the initial versions, identified, and resolved discrepancies, and created a harmonized prototype of the DSAS-2 Spa-US (15). In the third step, the third translator, a native English speaker, translated the harmonized prototype from Spanish into English. In the fourth step, the team presented the English translation of the harmonized prototype to a representative of the Australian team that developed the original English language DSAS-2, who liaised with the senior researcher on the Australian team. In this meeting, any translation challenges were discussed and resolved by consensus agreement. For example, there was some difficulty finding Spanish terms and phrases equivalent to the English terms and phrases used in the DSAS-2: “shame,” “I’m ashamed,” and “I feel embarrassed.” Although several possible Spanish terms and phrases were considered, it was decided, based on other widely used and well-respected healthcare resources that are translated from English into Spanish, to use the Spanish term “vergüenza” to translate the term “shame,” the Spanish phrase “me da vergüenza” to translate the English phrase “I’m ashamed,” and the Spanish phrase “me siento avergonzado/a” to translate the English phrase “I feel embarrassed” (16, 17).

In the fifth and sixth steps, the principal investigator conducted a focus group (April 2018) of Spanish-speaking community health workers (n=5) who had professional experience providing diabetes education and support in Spanish for Latino adults with T2D and then conducted cognitive debriefing interviews in Spanish (May 2018) with Spanish-speaking Latino adults with T2D (n=8), to elicit feedback on the prototype of the DSAS-2 Spa-US approved by the Australian team. The community health workers and the Latino adults with T2D were recruited from a health services organization serving communities in West Michigan. The cognitive debriefing interview participants were asked to reflect on and respond to each of the DSAS-2 Spa-US items and then share what they had thought about when reading and contemplating their responses to the DSAS-2 Spa-US items. The focus group participants each received a $50 gift card in appreciation of one hour of their time, while cognitive debriefing interviews participants each received a $25 gift card for 30 minutes of their time. The translation team reviewed the data for cases where participants consistently indicated that they had trouble with the instructions, the items, or the response options. Minor rewordings were discussed and agreed upon, changes were made. In the seventh and final step, two scientific experts in the field of diabetes-related psychosocial support who are both native Spanish speakers, one of Cuban heritage, and the other of Mexican heritage, assessed the DSAS-2 Spa-US for the use of appropriate language for Latino adults living with T2D in the U.S.

### Field testing and psychometric validation of the DSAS-2 Spa-US

In the second phase of the study, the DSAS-2 Spa-US was field-tested by Spanish-speaking U.S. Latino adults with T2D who participated in an online survey using Qualtrics XM software (Seattle, WA). The inclusion criteria were being 18 years or older, residing currently in the U.S., speaking Spanish, identifying as Hispanic or Latino, and having a current diagnosis of T2D. A total of 817 people responded to the survey, which was advertised through Facebook between October 2018 and June 2019. This study includes data on the 517 survey respondents who completed >90% of the 19-item DSAS-2 Spa-US. All participants provided consent before enrollment. Due to the anonymous nature of the online survey, participants did not receive compensation for their time, which might account for the large dropout before survey completion.

Participants provided sociodemographic and clinical information, including age, sex, country of origin, type of geographic area (urban/rural/suburban), educational level, relationship status, employment status, household income, diabetes duration, and diabetes treatment modality. Participants were asked to report their height and body weight to enable the calculation of body mass index (BMI) [weight (kg) divided by height (m^2^)].

Participants were administered Spanish-language versions of instruments measuring several psychological constructs, which are hypothesized to have relationships with diabetes stigma (7). A Spanish-language version of the 8-item Stigma Scale for Chronic Illnesses (SSCI-8; α = 0.84), measures general chronic illness stigma (18). Permission was granted for an instruction to be added before the items of the SSCI-8, which encouraged the respondents to respond regarding their diabetes (Jones, J. P., personal communication, June 1, 2018). A Spanish-language version (19) of the 17-item Diabetes Distress Scale (DDS-17; α = 0.96) measures diabetes-specific emotional distress (20). A Spanish-language version (21) of the 8-item Patient Health Questionnaire (PHQ-8; α = 0.89) measures depressive symptoms (22). A Spanish-language version (23) of the 7-item Generalized Anxiety Disorder-7 scale (GAD-7; α = 0.91), measures anxiety symptoms (24). A Spanish-language version (25) of the University of California Los Angeles (UCLA) 3-item loneliness scale (α = 0.87), measures loneliness (26). A Spanish-language version (27) of the Rosenberg Self-Esteem Scale (RSE; α = 0.79) measures general self-esteem (28).

### Data analysis

A descriptive approach was used to analyze the qualitative data (29). Univariate analyses were used to describe the characteristics of the study sample. Exploratory factor analysis (unrotated) was used to determine if the full set of the 19 items of the DSAS-2 Spa-US clustered together into one or more factors. Internal consistency reliability was assessed with Cronbach’s alpha, α. Following convention, simple imputation was applied in cases where <10% of data were missing for scoring the DSAS-2 Spa-US, the SSCI-8, the DDS, the PHQ-8, the GAD-7, and the RSE. Convergent validity was assessed against the scores of the SSCI-8, the DDS, the PHQ-8, the GAD-7, the 3-item UCLA Loneliness Scale, and the RSE. Based on the theoretical absence of a relationship between duration of diabetes and diabetes stigma, discriminant validity was assessed against diabetes duration. Moderate-to-large positive or negative correlations were expected as evidence of support of convergent validity. Correlations were considered, negligible (r_s_<0.10), small (r_s_≥0.10-0.29), moderate (r_s_≥0.30-0.49), or large (r_s_≥0.50) (30). A p-value of < 0.05 was considered statistically significant. Analyses were performed in STATA Version 16 (College Station, TX).

## Results

### Development of the DSAS-2 Spa-US

The primary issues raised by the community health worker participants in the focus group concerned the importance of the DSAS-2 Spa-US meeting the needs of individuals with varying levels of reading comprehension and health literacy. Based on the cognitive debriefing interview data, the translation of two items was identified as notable and needed further consideration. In the first case, the item stated in English reads, “Health professionals think that people with type 2 diabetes don’t know how to take care of themselves.” As it was translated into Spanish, it led some of the cognitive debriefing interview participants to interpret the phrase “don’t know how to” as “don’t know the ways” or “don’t know the information.” Thus, the item was interpreted as asking if it is perceived that health professionals think that people with diabetes need information and help with their self-care for their diabetes. According to the concept elaboration document provided by the Australian team, this contrasted with the original intent of the item, which was to ask whether it is perceived that healthcare professionals are generally unfair or unjustified in their judgments of individuals’ self-care of their diabetes. The second item that was identified for further consideration was “Because I have type 2 diabetes, some people judge me for my food choices.” Some of the cognitive debriefing interview participants took the interpretation of this item to mean that people had the desire for guests to eat what their hosts offered, in the sense that it would be rude or disrespectful not to eat what a host provided. Based on the explanation of the item in the concept elaboration document, this item was intended to convey a sense of judgment.

The focus group feedback was reviewed and discussed by the team, and their suggestions were incorporated into the DSAS-2 Spa-US. The team discussed the translation challenges that were uncovered in the cognitive debriefing interviews and decided to keep the translations of the two items as they were in the DSAS-2 Spa-US to maintain as much consistency as possible with the original English language DSAS-2. After minor rewording changes were made (**Appendix 1**) a consensus-derived determination was made that the final DSAS-2 Spa-US displayed the use of appropriate language linguistically and culturally for U.S. Latino adults living with diabetes.

### Psychometric properties of the DSAS-2 Spa-US in Latino adults with T2DM

The characteristics of the study sample of participants in the online survey are displayed in **Table 1**. The mean (SD) age was 54 (10) years old; 72% (n=374) were female, 54% (n=281) were born in Mexico, 62% (n=320) had an education level of high school or less, 51% (n=263) were not currently working, 71% (n=368) had a yearly household income of <$40,000, and 73% (n=375) were currently residing in urban areas. Fifty-eight percent (n=302) reported a height and body weight indicating a BMI ≥30 kg/m^2^, the mean (SD) duration of T2D was 10 (9) years, and 30% (n=156) used insulin to manage their T2D.

**Table 1:** Characteristics of study participants (*N*=517).

Age (years), mean ± *SD*	53.9 ± 10.1
Sex, female, *n* (%)	374 (72.3)
Education level, *n* (%)	
Primary school (grades 1-5)	50 (9.7)
Middle school (grades 6-8)	83 (16.1)
High school (grades 9-12)	187 (36.2)
Technical school or university	190 (36.8)
Employment status, *n* (%)	
Full-time	158 (30.6)
Part-time	84 (16.3)
Not working	263 (50.9)
Yearly household income, *n* (%)	
< $10,000	155 (30.0)
$10,000 to < $20,000	127 (24.6)
$20,000 to < $40,000	86 (16.6)
≥ $40,000	105 (20.3)
Don**’**t know/prefer not to say	44 (8.5)
Relationship status, *n* (%)	
Single	63 (12.2)
Married or living together	324 (62.6)
Divorced or separated or widowed	124 (24.0)
Place of birth, *n* (%)	
U.S. (50 states and District of Columbia)	40 (7.7)
Mexico	281 (54.4)
Caribbean	74 (14.3)
Central America	53 (10.3)
South America	42 (8.1)
Geographic area, *n* (%)	
Urban	375 (72.5)
Suburban	62 (12.0)
Rural	46 (8.9)
BMI category, *n* (%)	
Underweight (<18.5 kg/m^2^)	4 (0.8)
Normal weight (18.5-24.9 kg/m^2^)	52 (10.1)
Overweight (25-29.9 kg/m^2^)	159 (30.8)
Obesity (30-39.9 kg/m^2^)	191 (36.9)
Severe obesity (≥40 kg/m^2^)	111 (21.5)
Duration of diabetes diagnosis (years), Mean ± *SD* (range)	10.2 ± 9.4 (0-59)
Primary diabetes treatment, *n* (%)	
Lifestyle	35 (7.6)
Oral hypoglycemic agents	281 (61.2)
Insulin	156 (30.2)
Diabetes specific distress (DDS-17 score)	
Mean ± *SD*, (range)	2.9 ± 1.3 (1-6)
Median (25^th^ - 75^th^ percentile)	2.7 (1.6, 3.9)
Depressive symptoms (PHQ-8 score)	
Mean ± *SD*, (range)	7.3 ± 5.8 (0-24)
Median (25^th^ - 75^th^ percentile)	6.0 (3.0, 11.0)
Anxiety symptoms (GAD-7 score)	
Mean ± *SD*, (range)	6.1 ± 5.3 (0-21)
Median (25^th^ - 75^th^ percentile)	5.0 (2.0, 9.0)
Loneliness (UCLA 3-item loneliness scale)	
Mean ± *SD*, (range)	1.7 ± 0.6 (1-3)
Median (25^th^ - 75^th^ percentile)	1.7 (1.0 - 2.0)
Self-esteem (RSE score)	
Mean ± *SD*, (range)	31.1 ± 4.8 (15-40)
Median (25^th^ - 75^th^ percentile)	32.0 (28.0, 35.0)

BMI, Body Mass Index; DDS-17, Diabetes Distress Scale (17-items); GAD-7, Generalized Anxiety Disorder-7 scale (7-items); PHQ-8, Patient Health Questionnaire (8-items); RSE, Rosenberg Self-Esteem Scale; UCLA, University of California Los Angeles.

a Frequencies do not always add to N=517 due to missing data on some items.

**Figure 1:**
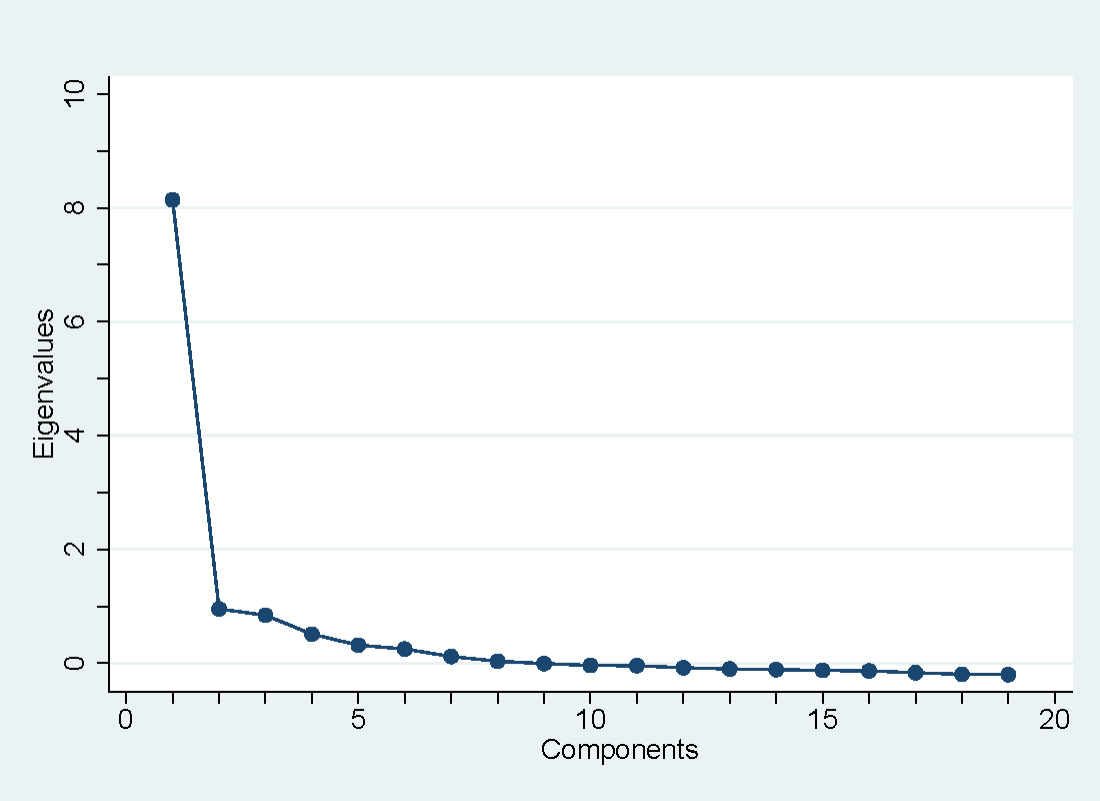
Scree plot.

Exploratory factor analyses revealed that 82% of the variance in the participant responses to the 19 items of the DSAS-2 Spa-US was explained by a single factor (**Table 2**), with all the items loading >0.5. The scree plot also suggested a single factor because the eigenvalues level off after one factor (**Figure 1**). A three-factor solution was not apparent, suggesting that subscale scores are not supported for the DSAS-2 Spa-US. The internal reliability of the total DSAS-2 Spa-US scale was high (α=0.93), supporting the calculation of a total DSAS-2 Spa-US score representing diabetes stigma. For all 19 items, the full range of response options was endorsed. The frequency distributions of the individual items had consistently positive skews, ranging from 0.21 to 1.45. The kurtosis for individual items was also consistently positive, ranging from 1.56 to 4.70. Missing response data for the individual items ranged from 1% to 4% (**Table 3**).

**Table 2:** Factor loadings and unique variances based on a principal components analysis (unrotated) for the 19 items of the DSAS-2 Spa-US (N=517).

Item wording and Spanish language translation	Single-factor solution
Some people think I cannot fulfill my responsibilities (e.g., work, family) because I have type 2 diabetes *Algunas personas piensan que no puedo cumplir con mis responsabilidades (por ejemplo: trabajo, familia) porque tengo diabetes tipo 2*	0.52
Some people treat me like I’m “sick” or “ill” because I have type 2 diabetes *Algunas personas me tratan como si estuviera “enfermo/a” porque tengo diabetes tipo 2*	0.58
Some people see me as a lesser person because I have type 2 diabetes *Algunas personas me ven como una persona inferior porque tengo diabetes tipo 2*	0.74
Some people exclude me from social occasions that involve food/drink they think I shouldn’t have *Algunas personas me excluyen de eventos sociales donde hay comidas o bebidas que piensan que no debo comer o beber*	0.70
I have been discriminated against in the workplace because of my type 2 diabetes *Me han discriminado en el trabajo por tener diabetes tipo 2*	0.56
I have been rejected by others (e.g., friends, colleagues, romantic partners) because of my type 2 diabetes *He sido rechazado/a por otras personas (por ejemplo: amigos, colegas, parejas) porque tengo diabetes tipo 2*	0.66
I have been told that I brought my type 2 diabetes on myself *Me han dicho que yo he causado mi diabetes tipo 2*	0.64
There is blame and shame surrounding type 2 diabetes *Hay culpa y vergüenza con la diabetes tipo 2*	0.72
Because I have type 2 diabetes, some people judge me for my food choices *Porque tengo diabetes tipo 2, algunas personas me juzgan por las comidas que escojo*	0.59
Health professionals think that people with type 2 diabetes don’t know how to take care of themselves *Algunos profesionales de la salud piensan que las personas con diabetes tipo 2 no saben cuidarse*	0.53
Because of my type 2 diabetes, health professionals have made negative judgments about me *Porque tengo diabetes tipo 2, algunos profesionales de la salud me han juzgado de manera negativa*	0.59
There is a negative stigma about type 2 diabetes being a “lifestyle disease” *La diabetes tipo 2 tiene un estigma negativo por ser una enfermedad de ‘estilo de vida’*	0.65
Because I have type 2 diabetes, some people assume I must be overweight, or have been in the past *Porque tengo diabetes tipo 2, algunas personas suponen que tengo sobrepeso o que he tenido sobrepeso en el pasado*	0.54
I feel embarrassed in social situations because of my type 2 diabetes *Me siento avergonzado/a por mi diabetes tipo 2*	0.72
I’m ashamed of having type 2 diabetes *Me da vergüenza tener diabetes tipo 2*	0.73
I blame myself for having type 2 diabetes *Me culpo a mí mismo/a por tener diabetes tipo 2*	0.64
Because I have type 2 diabetes, I feel like I am not good enough *Porque tengo diabetes tipo 2, siento que no soy lo suficientemente bueno/a*	0.73
Having type 2 diabetes makes me feel like a failure *Tener diabetes tipo 2 me hace sentir como un fracaso*	0.79
I feel guilty for having type 2 diabetes *Me siento culpable por tener diabetes tipo 2*	0.71
**Eigenvalue**	8.14
**% Variance explained**	81.55
**Cronbach alpha**	0.93

DSAS-2 Spa-US: Type 2 Diabetes Stigma Assessment Scale Spanish language translation.

**Table 3:** DSAS-2 Spa-US item response patterns and missing data (N=517).

	Response options					
Items	Strongly disagree	Disagree	Unsure	Agree	Strongly agree	Missing data
Some people think I cannot fulfill my responsibilities (e.g., work, family) because I have type 2 diabetes	285 (55.1)	112 (21.7)	62 (12.0)	27 (5.2)	23 (4.4)	8 (1.5)
Some people treat me like I’m “sick” or “ill” because I have type 2 diabetes	225 (43.5)	103 (19.9)	80 (15.5)	78 (15.1)	24 (4.6)	7 (1.4)
Some people see me as a lesser person because I have type 2 diabetes	294 (56.9)	92 (17.8)	74 (14.3)	27 (5.2)	18 (3.5)	12 (2.3)
Some people exclude me from social occasions that involve food/drink they think I shouldn’t have	291 (56.3)	97 (18.8)	69 (13.3)	37 (7.2)	15 (2.9)	8 (1.5)
I have been discriminated against in the workplace because of my type 2 diabetes	299 (57.8)	84 (16.2)	84 (16.2)	14 (2.7)	16 (3.1)	20 (3.9)
I have been rejected by others (e.g., friends, colleagues, romantic partners) because of my type 2 diabetes	307 (59.4)	101 (19.5)	81 (15.7)	9 (1.7)	11 (2.1)	8 (1.5)
I have been told that I brought my type 2 diabetes on myself	214 (41.4)	88 (17.0)	71 (13.7)	90 (17.4)	49 (9.5)	5 (1.0)
There is blame and shame surrounding type 2 diabetes	250 (48.4)	106 (20.5)	61 (11.8)	61 (11.8)	28 (5.4)	11 (2.1)
Because I have type 2 diabetes, some people judge me for my food choices	202 (39.1)	71 (13.7)	88 (17.0)	107 (20.7)	41 (7.9)	8 (1.5)
Health professionals think that people with type 2 diabetes don’t know how to take care of themselves	179 (34.6)	77 (14.9)	70 (13.5)	125 (24.2)	60 (11.6)	6 (1.2)
Because of my type 2 diabetes, health professionals have made negative judgments about me	246 (47.6)	101 (19.5)	102 (19.7)	35 (6.8)	20 (3.9)	13 (2.5)
There is a negative stigma about type 2 diabetes being a “lifestyle disease”	201 (38.9)	101 (19.5)	83 (16.1)	85 (16.4)	39 (7.5)	8 (1.5)
Because I have type 2 diabetes, some people assume I must be overweight, or have been in the past	178 (34.4)	91 (17.6)	81 (15.7)	100 (19.3)	59 (11.4)	8 (1.5)
I feel embarrassed in social situations because of my type 2 diabetes	290 (56.1)	97 (18.8)	49 (9.5)	49 (9.5)	26 (5.0)	6 (1.2)
I’m ashamed of having type 2 diabetes	286 (55.3)	95 (18.4)	49 (9.5)	50 (9.7)	23 (4.5)	14 (2.7)
I blame myself for having type 2 diabetes	211 (42.7)	71 (13.7)	56 (10.8)	109 (21.1)	47 (9.1)	13 (2.5)
Because I have type 2 diabetes, I feel like I am not good enough	287 (55.5)	115 (22.2)	55 (10.6)	32 (6.2)	20 (3.9)	8 (1.5)
Having type 2 diabetes makes me feel like a failure	255 (49.3)	112 (21.7)	68 (13.2)	49 (9.5)	26 (5.0)	7 (1.4)
I feel guilty for having type 2 diabetes	266 (51.5)	72 (13.9)	47 (9.1)	85 (16.4)	42 (8.1)	5 (1.0)

DSAS-2 Spa-US, Type 2 Diabetes Stigma Assessment Scale U.S. Spanish language U.S. translation.

Convergent validity was demonstrated with strong, positive correlations observed between diabetes stigma and general chronic illness stigma (r_s_=0.65, p<0.001) and diabetes distress (r_s_=0.57, p<0.001), and medium, positive correlations observed between diabetes stigma and depressive symptoms (r_s_=0.45, p<0.001), anxiety symptoms (r_s_=0.43, p<0.001), and loneliness (r_s_=0.41, p<0.001). There was a medium, negative correlation between diabetes stigma and general self-esteem (r_s_=-0.51, p<0.001). Discriminant validity was supported by the lack of correlation between diabetes stigma and duration of diabetes (r=0.07, p=0.120).

## Discussion

The DSAS-2 was translated into Spanish, and the field test results of the DSAS-2 Spa-US among U.S. Latino adults with T2D indicate good psychometric performance. The factor analysis identified a single factor (explaining 82% of the variance) with strong internal consistency reliability. Evidence of the convergent and divergent validity of the DSAS-2 Spa-US was demonstrated, confirming the hypothesized relationships between diabetes stigma and general chronic illness stigma, diabetes distress, depressive and anxiety symptoms, loneliness, and general self-esteem. The lack of association between diabetes stigma and duration of diabetes demonstrated evidence of discriminant validity.

The psychometric performance of the DSAS-2 Spa-US among U.S. Latino adults with T2D differed somewhat from that of the DSAS-2 among Australian adults with T2D. In the current study, there was strong support for a single unidimensional scale but no evidence for the three subscales (Treated Differently, Blame and Judgement, and Self-Stigma), which were identified during the original validation of the DSAS-2 among Australian adults with T2D (7). This discrepancy may be due to the Australian participants having a higher education level than the U.S. Latino participants (60% versus 37% with more than a high school education). Though it is unclear whether the differing factor structure can be attributed to education, it is important since adults with higher educational levels are more likely to report experiences and perceptions of health-related stigma (4, 31). Another possible explanation is that despite the authors’ efforts, some concepts were challenging to translate from English into Spanish. A recently published study, which examined the psychometric properties of the DSAS-2 Spa-US using data from adults with T2D in Colombia, also provides evidence of support for a single unidimensional scale (32). Taken together, the findings highlight the need for further attention to education, culture, and race/ethnicity in diabetes stigma research, as there may be differences in the ways that having T2D is construed and in the value that is placed on orientation toward others and to the self that results in diabetes stigma being enacted differently. For now, these findings suggest that there is no support for the three subscales of the DSAS-2, and that only the total DSAS-2 scale score should be calculated when using the DSAS-2 Spa-US in Spanish-speaking adults with T2D in the US and in Colombia.

Latino adults in the U.S. constitute a large and diverse ethnic group, although there are commonalities, including using Spanish as a shared language (33). To an extent, the diversity is reflected in the backgrounds of the participants in the study sample, in which adults with Mexican heritage accounted for the largest proportion of the study participants in the online survey (54%). Although for Spanish-speaking groups, there are differences among the languages spoken in different parts of the country, the multiphase process that was used to develop the DSAS-2 Spa-US was intended to capture the true meaning of the English language used in the DSAS-2 to produce a Spanish-language translation of the DSAS-2 of high-quality and integrity.

### Implications for practice and future research

The DSAS-2 Spa-US can be used to assess diabetes stigma in Latino U.S. adults with T2D as part of future studies to determine the impact of diabetes stigma on clinical and psychosocial outcomes, as well as to examine the impact of interventions designed to minimize diabetes stigma. Studies using the DSAS-2 in adults with T2D in the U.S. indicate that diabetes stigma is associated with higher diabetes distress, lower engagement in diabetes self-management, lower diabetes self-efficacy, and lower quality interactions with healthcare professionals (9). Studies to determine if similar relationships exist between diabetes stigma and these constructs using the DSAS-2 Spa-US in Latino U.S. adults with T2D are needed.

### Limitations

This study has some limitations. Due to the cross-sectional nature of the study, we could not determine the test-retest reliability of the DSAS-2 Spa-US (i.e., how consistently individuals might respond if asked to repeat the DSAS-2 Spa-US within a short period). Evidence supporting the validation of inferences made with the DSAS-2 Spa-US may have been stronger if test-retest reliability was evaluated. However, longitudinal stigma studies are lacking, and the trajectory of stigma over time is unknown. Nor could we determine the predictive validity of the DSAS-2 Spa-US (i.e., the extent to which diabetes stigma at baseline predicts future clinical or psychosocial outcomes). The DSAS-2 Spa-US was used in a large sample in which participants self-reported their clinical data. Using the DSAS-2 Spa-US in a clinical setting where clinical data could be obtained from electronic medical records would be desirable. The responsiveness of the DSAS-2 Spa-US in Latino adults with T2D will need to be examined in future intervention studies. The generalizability of the current research is dependent on the sample being representative of U.S. Latino adults with T2D. Participants were recruited *via* a social media platform and completed the survey online using a smartphone, a desktop, a laptop, or a tablet computer. While there have been gains in technology adoption in the U.S., the ‘digital divide’ still exists, which may have created barriers to participation for Latino adults with T2D who are older, have lower incomes, and live in rural areas (34, 35).

### Future research

This study contributes to the public health imperative to address diabetes disparities experienced among ethnic minority communities in the U.S. with the development of the DSAS-2 Spa-US to measure diabetes stigma in Latino adults with T2D. Experiences and perceptions of diabetes stigma remain understudied among adults with T2D. Considering the high prevalence of T2D among Latino adults in the U.S., research needs to continue to examine the effects of diabetes stigma on Latino populations’ diabetes self-management and overall well-being.

### Conclusion

This study demonstrates that the DSAS-2 Spa-US is a valid and reliable assessment of diabetes stigma and is suitable for U.S. Latino adults with T2D. It is ready for use in research to examine the experience and impact of diabetes stigma in U.S. Latino adults with T2D, an underrepresented ethnic group, to accelerate health equity and eliminate disparities in diabetes health outcomes and health care.

## Data availability statement

The raw data supporting the conclusions of this article will be made available by the authors, without undue reservation.

## Ethics statement

The studies involving human participants were reviewed and approved by The University of Michigan Institutional Review Board. The patients/participants provided their written informed consent to participate in this study.

## Author contributions

Conceptualization, KJ and JS. Methodology, KJ and JS. Formal analysis and investigation, KJ and AB. Writing - original draft preparation, KJ and MA. Writing - review and editing, JS. Funding acquisition, JS. Resources, KJ and JS. Supervision, JS. All authors contributed to the article and approved the submitted version.
