# Supplementary material for: A Spanish-language translation for the U.S. of the type 2 diabetes stigma assessment scale (DSAS-2 Spa-US)
Source: Front Clin Diabetes Healthc. 2022 Dec 23;3:1057559. doi: 10.3389/fcdhc.2022.1057559 (PMC10012130; doi:10.3389/fcdhc.2022.1057559)
Supplement: Supplementary file 1 [file Table_1.docx]

**DSAS-2 Spa-US. Development Details**

| **Original DSAS-2** | **DSAS-2 Spa-US presented to the scientific experts** | **Final DSAS-2 Spa-US based on scientific experts' recommendations** |
| --- | --- | --- |
| Some people think I cannot fulfill my responsibilities (e.g. work, family) because I have type 2 diabetes | Algunas personas piensan que no puedo cumplir con mis responsabilidades (por ejemplo: trabajo, familia) porque tengo diabetes tipo 2 | Not modified |
| Some people treat me like I'm "sick" or "ill" because I have type 2 diabetes | Algunas personas me tratan como si estuviera “enfermo/a” porque tengo diabetes tipo 2 | Not modified |
| Some people see me as a lesser person because I have type 2 diabetes | Hay gente que me ve como una persona inferior porque tengo diabetes tipo 2 | Algunas personas me ven como una persona inferior porque tengo diabetes tipo 2 |
| Some people exclude me from social occasions that involve food /drink they think I shouldn't have | Algunas personas me excluyen de los eventos sociales donde hay comidas o bebidas que piensan que no debo comer o beber | Algunas personas me excluyen de eventos sociales donde hay comidas o bebidas que piensan que no debo comer o beber |
| I have been discriminated against in the workplace because of my type 2 diabetes | Me han discriminado en mi trabajo por tener diabetes tipo 2 | Me han discriminado en el trabajo por tener diabetes tipo 2 |
| I have been rejected by others (e.g., friends, colleagues, romantic partners) because of my type 2 diabetes | He sido rechazado/a por otras personas (por ejemplo: amigos, colegas, parejas) porque tengo diabetes tipo 2 | Not modified |
| I have been told that I brought my type 2 diabetes on myself | Me han dicho que yo mismo causé mi diabetes tipo 2 | Me han dicho que yo he causado mi diabetes tipo 2 |
| There is blame and shame surrounding type 2 diabetes | La diabetes tipo 2 implica culpa y vergüenza | Hay culpa y vergüenza con la diabetes tipo 2 |
| Because I have type 2 diabetes, some people judge me for my food choices | Debido a la diabetes tipo 2, algunas personas me juzgan por las comidas que escojo | Porque tengo diabetes tipo 2, algunas personas me juzgan por las comidas que escojo |
| Health professionals think that people with type 2 diabetes don't know how to take care of themselves | Los profesionales de la salud piensan que la gente con diabetes tipo 2 no sabe cuidarse | Algunos profesionales de la salud piensan que las personas con diabetes tipo 2 no saben cuidarse |
| Because of my type 2 diabetes, health professionals have made negative judgements about me | Algunos profesionales de la salud me han juzgado de manera negativa porque tengo diabetes tipo 2 | Porque tengo diabetes tipo 2, algunos profesionales de la salud me han juzgado de manera negativa |
| There is a negative stigma about type 2 diabetes being a “lifestyle disease” | La diabetes tipo 2 tiene un estigma negativo por ser una enfermedad causada por el estilo de vida | La diabetes tipo 2 tiene un estigma negativo por ser una enfermedad de ‘estilo de vida’ |
| Because I have type 2 diabetes, some people assume I must be overweight, or have been in the past | Porque tengo diabetes tipo 2, algunas personas suponen que tengo sobrepeso o que he tenido sobrepeso en el pasado | Not modified |
| I feel embarrassed in social situations because of my type 2 diabetes | Me siento incomodo o cohibido por tener diabetes tipo 2 | Me siento avergonzado/a por mi diabetes tipo 2 |
| I'm ashamed of having type 2 diabetes | Me siento avergonzado de tener diabetes tipo 2 | Me da vergüenza tener diabetes tipo 2 |
| I blame myself for having type 2 diabetes | Me culpo a mí mismo/a por tener diabetes tipo 2 | Not modified |
| Because I have type 2 diabetes, I feel like I am not good enough | Siento que no soy lo suficientemente bueno porque tengo diabetes tipo 2 | Porque tengo diabetes tipo 2, siento que no soy lo suficientemente bueno/a |
| Having type 2 diabetes makes me feel like a failure | Tener diabetes tipo 2 me hace sentir como un fracaso | Not modified |
| I feel guilty for having type 2 diabetes | Me siento culpable por tener diabetes tipo 2 | Not modified |
